# Machine Learning Scoring Reveals Increased Frequency of Falls Proximal to Death in *Drosophila melanogaster*

**DOI:** 10.1093/gerona/glaf029

**Published:** 2025-02-15

**Authors:** Faerie Mattins, Shriya Nagrath, Yijie Fan, Tomás Kevin Delgado Manea, Shoham Das, Aditi Shankar, John Tower

**Affiliations:** Molecular and Computational Biology Section, Department of Biological Sciences, University of Southern California, Los Angeles, California USA; Molecular and Computational Biology Section, Department of Biological Sciences, University of Southern California, Los Angeles, California USA; Molecular and Computational Biology Section, Department of Biological Sciences, University of Southern California, Los Angeles, California USA; Molecular and Computational Biology Section, Department of Biological Sciences, University of Southern California, Los Angeles, California USA; Molecular and Computational Biology Section, Department of Biological Sciences, University of Southern California, Los Angeles, California USA; Molecular and Computational Biology Section, Department of Biological Sciences, University of Southern California, Los Angeles, California USA; Molecular and Computational Biology Section, Department of Biological Sciences, University of Southern California, Los Angeles, California USA; (Biological Sciences Section)

**Keywords:** Aging, Computer vision, Dehydration stress, YOLO

## Abstract

Falls are a significant cause of human disability and death. Risk factors include normal aging, neurodegenerative disease, and sarcopenia. *Drosophila melanogaster* is a powerful model for study of normal aging and for modeling human neurodegenerative disease. Aging-associated defects in *Drosophila* climbing ability have been observed to be associated with falls, and immobility due to a fall is implicated as one cause of death in old flies. An automated method for quantifying *Drosophila* falls might facilitate the study of causative factors and possible interventions. Here, machine learning methods were developed to identify *Drosophila* falls in video recordings of 2D movement trajectories. The study employed existing video of aged flies as they approached death, and young flies subjected to lethal dehydration/starvation stress. Approximately 9 000 frames of video were manually annotated using open-source tools and used as the training set for You Only Look Once (YOLOv4) software. The software was tested on specific hours within a 22 hour video that was originally manually annotated for number of falls per hour and corresponding timestamps. The model predictions were evaluated against the manually-annotated ground truth, revealing a strong correlation between the predicted and actual falls. The frequency of falls per hour increased dramatically 2–4 hours prior to death caused by dehydration/starvation stress, whereas extended periods of increased falls were observed in aged flies prior to death. This automated method effectively quantifies falls in video data without observer bias, providing a robust tool for future studies aimed at understanding causative factors and testing potential interventions.

Falls are a major worldwide health issue, with limited available treatments or interventions. Risk factors include normal aging as well as neurodegenerative disease and sarcopenia (1,2). In U.S. adults age 65 and older, 36 million falls occur each year. Approximately 20% of these falls result in serious injury, including traumatic brain injury (TBI) and hip fractures, and falls were the most common cause of TBI-related deaths (3,4). Interventions for aging-associated falls in humans are currently limited to exercise, and therefore an effective dietary or small-molecule intervention would have significant implications for improving human health and quality of life.

*Drosophila* is major research model for normal aging and for neurodegenerative disease, including human neurodegenerative diseases associated with increased falls, such as Parkinson’s disease, Friedreich’s ataxia, and Huntington’s disease (5–11). Several studies report analysis of 2D and 3D *Drosophila* movement behaviors during aging, using video (12–17). These video-based approaches reveal aging-associated deteriorations in behaviors, climbing speed, and total movement activity. Aging-associated defects in *Drosophila* climbing ability have been observed to be associated with falls (15,16), however, fall frequency has not been specifically quantified. Previous 3D tracking of fly movement indicated that the majority of flies exhibit episodes of erratic movement, as indicated by increased frequency of directional heading changes (FDHC), in the 8 hours prior to death due to aging or dehydration/starvation stress (16). The erratic movement was observed to be associated with falls, often to a supine (on the back) position. In one life span assay, 42% of flies were found dead in the supine position, consistent with the idea that inability to right themselves after a fall might be a common cause of death for *Drosophila* (18).

Here we develop a machine learning tool for quantification of falls in video recordings of 2D *Drosophila* movement behaviors. Video segments corresponding to manually scored falls were used as training data for a machine learning model, to enable automated scoring and quantification of falls without potential observer bias. The long-term goal of these studies is to provide increased understanding of the underlying mechanisms of aging-associated falls, and facilitate future screens for interventions with potential applications to human health.

## Method

### Data set

**Figure 1.**
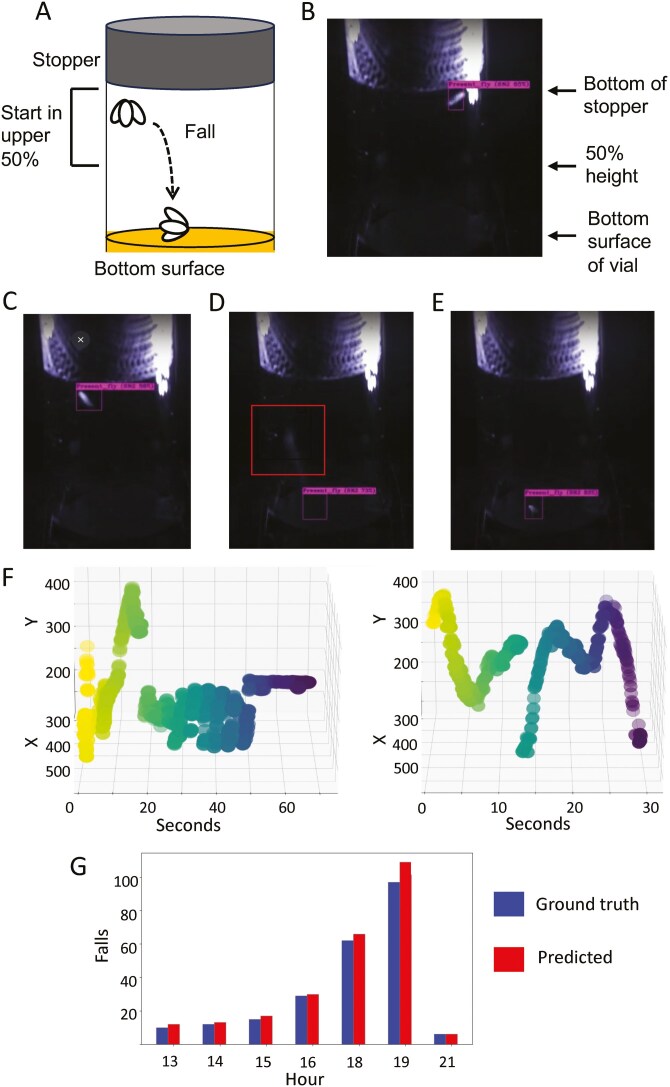
Scoring fly falls. (**A**) Falls assay. The fly is housed in a standard glass vial containing media, with a black cloth stopper. The operational criteria for scoring a fall is as follows: the fly begins in a standing position on the surface of the vial, located in the upper 50% of the available area, and rapidly moves directly to the bottom surface. (**B–E**) YOLO scoring of a fly fall. (**B**) The software correctly identifies the fly, as indicated by the labelled bounding box. This includes distinguishing the fly from the background glare caused by the stopper. (**C**) The fly begins in a standing position on the vial surface, as recognized by the software. (**D**) The fly moves rapidly towards the bottom surface, as indicated by a faint white streak in the center region of the video frame (indicated by square box). This rapid downward movement is too fast for the fly to be continuously detected by the software, and the fly is next detected on the bottom surface of the vial. (**E**) Fly detected on the bottom surface of the vial. (**F**) Example plots of detected fly falls. The trajectory generated by YOLO tracking is presented. The plot shows the position of the fly on the vertical axis as a function of time in seconds, immediately before and after a fall. The fall is indicated by a characteristic gap in the tracking trajectory, which begins at a high position on the vertical axis and ends at a low position on the vertical axis. (**G**) Analysis of the test data. A single *w[1118]* strain male, age 3-5 days was placed in an empty vial and video was continuously recorded using reflected IR light until the fly died in hour 22 (FLYID#05-26-19). Seven hours of the test data were selected for analysis. The number of ground truth falls are plotted in blue (left bars), and the number of falls predicted by YOLOv4 are indicated in red (right bars).

An existing video data set was used to analyze fly falls (16). The fly genotypes were *w[1118]* strain, and *w[1118]; 3xP3-GFP[M1]* strain, as previously described. To generate the video data, individual flies were placed in glass culture vials, which were then placed in a dark box illuminated with infrared (IR) light, and video was recorded at 30fps. Single young flies (3–14 days of age) were assayed in empty vials until they died from dehydration/starvation stress. Single aged flies (64–75 days of age), were placed in vials with media, video was recorded until death, and the last 48 hours of video containing the hour of death was analyzed. Death was defined as the absence of movement, including twitching of the legs. The criteria for a fall was a fly beginning in a standing position on the internal surface of the vial, located in the upper 50% of the available area, and rapidly falling directly to the bottom surface of the vial (Figure 1A). The distance from the bottom of the stopper to the top of the food, or to the bottom of the empty vial is adjusted to 3 cm in each case, by adjusting the position of the stopper. The time courses of fall frequency generated here are aligned with previously calculated time courses of total movement activity, frequency of directional heading changes (FDHC), and rate of change in FDHC (16), and are presented in the Supplementary Material with permission from Elsevier.

## YOLOv4 Configuration

To enable automated detection of fly falls in the videos, the YOLOv4 software was employed (19). YOLO is a real-time object detection system designed for computer vision and deep learning applications. It provides a single-pass detection method, processing an entire image in one sweep through a convolutional neural network (CNN). YOLO detects objects and provides precise bounding box coordinates for each detected object, along with class labels and associated confidence scores. Unlike other methods, YOLO does not rely on predefined anchors or region proposals; instead, it treats object detection as a regression task. YOLO has been trained on the Common Objects in Context (COCO) data set, which contains a large number of images with annotations for 80 different object categories, making it suitable for general object detection tasks. However, the COCO data set does not include images or categories for insects, making the publicly available version of YOLO unsuitable for analyzing *Drosophila* falls. Therefore, further training of YOLO was required using annotated data specific to *Drosophila* falls.

## Training Data

The training data was previously generated using 2 young *w[1118]* strain female flies subjected to dehydration/starvation stress (16). Individual flies were placed in empty vials, and video was continuously recorded using reflected IR light until the flies died. One female was 14 days old and died in hour 25 (FLYID#06-13-19), whereas the second female was 3–7 days old and died in hour 19 (FLYID#06-15-19). To annotate the training data set, frames in the videos corresponding to a fly fall were labeled by the annotator using Open Labeling (20). Open Labeling is a highly customizable open-source tool written in Python that enables the annotation of video data by drawing bounding boxes around objects of interest. These bounding boxes specify the class as well as the coordinates of the fly in each relevant frame. A detailed annotator guideline, assigning primary and secondary annotators, was used to guide the process. The reliability of the annotations was analyzed using Cohen’s kappa coefficient (21), and any videos where the value was less than 0.75 were omitted from the analysis. A total of 18 000 files were used to fine-tune the YOLOv4 model, consisting of 9 000 image files and 9 000 text files describing the bounding box coordinates. Approximately 90 total person hours were required to annotate the training data set. In total, there were five falls in the training set. The YOLOv4 model was trained using specific hyperparameters to optimize performance (Supplementary Material, Table 1). All software and associated files are available free for download from GitHub, with repository title “Fall-Prediction” (**https://github.com/johntower/Fall-Prediction**.

## Testing Data

The video data used for testing the software was previously generated using a single *w[1118]* strain male, age 3–5 days (16). The fly was placed in an empty vial and video was continuously recorded using reflected IR light until the fly died in hour 22 (FLYID#05-26-19). Here, researchers manually annotated specific hours within the 22 hour period to count the number of falls per hour and recorded their timestamps. Approximately 10 total person hours were required to annotate the testing data set. This “ground truth” testing data was not used for training the model. To evaluate the model predictions relative to the ground truth generated by manual annotation, the frequency of false positives and false negatives was calculated. An accurate scoring of a fall was defined as a model timestamp within ±1 second of the ground truth timestamp.

Little preparation time is required when recording videos, so recording a one hour video takes approximately one hour and 5 minutes. The Google Colab Pro environment was used to conduct video processing because this tool is relatively user-friendly. The time required for video processing is approximately equal to the length in time of the video. In addition to providing the number of falls and their timestamps, the software also provides a value for the total distance moved by the fly in pixels, calculated as total movement = sqroot((delta_x)^2 + (delta_y)^2). This allows for normalization of falls to total movement activity. Finally, the software allows for a threshold value to be set for the total distance in pixels that the fly must move in the Y direction to be scored as a fall.

## Results

### Detection of Fly Falls by the Model

An example of a fall detected by the software is presented (Figure 1B–E). The software distinguishes the fly from the background glare caused by the stopper, and correctly identifies the fly as it walks, as indicated by the bounding box (Figure 1B). The fly begins the fall sequence in a standing position on the interior vial surface, as recognized by the software (Figure 1C). The fly then moves rapidly toward the bottom surface, as indicated by a faint white streak in the center region of the video frame (Figure 1D; indicated by red box). This rapid downward movement is too fast for the fly to be continuously detected by the software, and the fly is next detected on the bottom surface of the vial (Figure 1E). Plotting the movement of the fly as detected by the software further illustrates the fall events (Figure 1F). The plots show the position of the fly on the vertical axis as a function of time in seconds, immediately before and after a fall. The fall is indicated by a characteristic gap in the tracking trajectory, which begins at a high position on the vertical axis and ends at a low position on the vertical axis.

## Testing the Accuracy of the Model

To test the accuracy of the falls scoring, the testing data set was analyzed. The recorded timestamps generated by manual annotations, which serve as the ground truth, were compared with the predicted timestamps generated by the YOLOv4 model, for selected hours of the testing data set (Table 1, Figure 1G). For the initial analysis, the threshold for the distance the fly must move in the Y direction to be scored as a fall was set at 60 pixels. The model demonstrated a high level of accuracy, closely matching the ground truth. Across 7 hours analyzed, the model predicted a total of 253 falls. Among these predicted falls were a total of 24 potential false positives (9.5%), and a total of 2 false negatives (0.8%). However, subsequent analysis of the potential false positives revealed that 21 were indeed falls, but that they began from a position below the 50% height criteria used for the manual annotation of the ground-truth data. The 3 cm distance between the stopper and the bottom surface of vial is estimated to correspond to 325 pixels. Increasing the Y direction threshold to 160 pixels eliminated the events that began below the 50% height criteria, and the frequency of actual false positives was then reduced to 3/253, or 1.2% (Table 1). Details of the predicted and ground truth timestamps for each hour of the test data are presented in the Supplementary Material (Supplementary Tables 2-8).

**Table 1.** Ground Truth Versus Predicted Falls (FLYID#05-26-19)

Hour #	Predicted Falls	Ground Truth Falls	Potential False Positives	Actual False Positives	False Negatives
Hour 13	12	10	2	1	0
Hour 14	13	12	1	0	0
Hour 15	17	15	2	0	0
Hour 16	30	29	2	0	1
Hour 18	66	62	4	0	0
Hour 19	109	97	13	2	1
Hour 21	6	6	0	0	0

## Increased Frequency of Falls Proximal to Death

**Figure 2.**
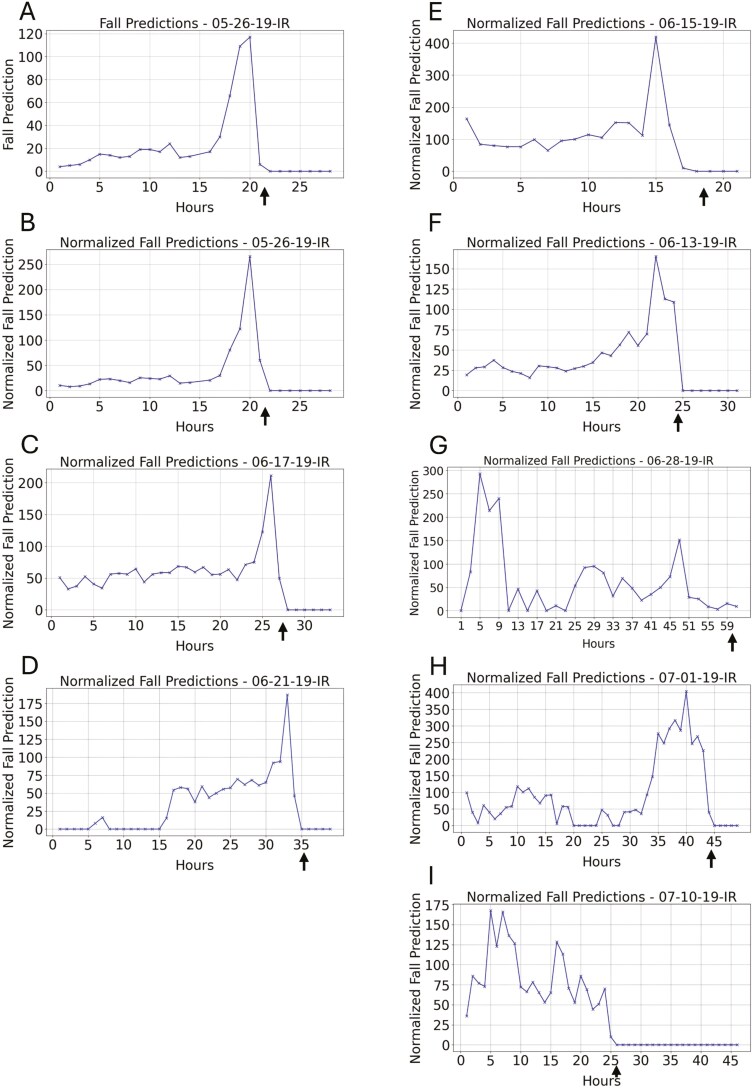
Increased fly falls proximal to death. YOLOv4 was used to score falls per hour. Arrows indicate the hour of fly death. (**A–F**) Flies in empty vials dying from dehydration/starvation stress. (**G–I**) Aged flies in vials with normal media. (**A,B**) Falls per hour for a young (3–5 day old) *w[1118]* male fly (FLYID#5-26-19). (**A**) Raw falls per hour. (**B**) Normalized falls per hour. Normalization was relative to total movement activity. (**C**) Normalized falls per hour for a young (1 day old) *w[1118]* female fly (FLYID#06-17-19). (**D**) Normalized falls per hour for a young (1 day old) *w[1118]* female fly (FLYID#06-21-19). **(E)** Normalized falls per hour for a young (3-7 day old) *w[1118]* female fly (FLYID#6-15-19). (**F**) Normalized falls per hour for a young (14 day old) *w[1118]* female fly (FLYID#06-13-19). (**G**) Normalized falls per hour for an aged (64 day old) *w[1118]* male fly (FLYID#06-28-19). (**H**) Normalized falls per hour for an aged (66 day old) *w[1118]* male fly (FLYID#07-01-19). **(I)** Normalized falls per hour for an aged (75 day old) *3xP3-GFP* male fly (FLYID#07-10-19).

When a single young fly is placed in an empty vial, it dies in less than 48 hours due to dehydration/starvation stress (16). Data were analyzed for a young (3–5 day old) *w[1118]* male fly, which dies in hour 22 (FLYID#05-26-19; Figure 2A). The model was used to score the number of falls per hour, which was observed to increase dramatically over a period of approximately three hours prior to death. To effectively evaluate possible changes in the frequency of falls per hour, it is important to account for any differences in total fly movement per hour, because increased fly movement is likely to increase frequency of falls. To do this, falls per hour were normalized to total fly movement per hour, to yield falls per hour per relative unit of fly movement. Normalizing the data in this way indicates a spike in falls frequency in the 2 hours prior to death (Figure 2B). An alignment of the fall frequency per hour versus total activity per hour is presented in the Supplementary Material (Supplementary Figure 1A). The same analysis was conducted for 4 additional young flies that died from dehydration/starvation stress, and in each case frequency of falls was observed to spike over a period of 2–4 hours immediately prior to death (Figure 2C–F). Notably, for each of the 5 flies, the spikes in fall frequency calculated here are found to correspond to periods of greatly decreased total movement, but increased erratic movement, as previously identified by calculating the FDHC (Supplementary Figure 1A–E).

Finally, fall frequency was assayed for 3 aged flies as they died while maintained on normal media. A single *w[1118]* strain male, aged 64 days, was assayed over 61 hours, and died in hour 60 (FLYID#06-28-19; Figure 2G). The frequency of falls was observed to spike at approximately 55 hours prior to death, and at approximately 5 hours prior to death. Similarly, a single *w[1118]* strain male, aged 66 days, was assayed over 48 hours, and died in hour 45 (FLYID#07-01-19; Figure 2H). The frequency of falls was observed to peak approximately 5 hours prior to death. Finally, a single *3xP3-GFP* strain male, aged 75 days, was assayed over 48 hours, died in hour 26, and exhibited an extended period of increased falls across approximately 20 hours prior to death (Figure 2I). For the aged flies, the periods of increased fall frequency are found to correspond to periods of increased total movement, as well as increased erratic movement (Supplementary Figure 2A–C).

## Discussion

*Drosophila* is a powerful model for analysis of behavior, including video and machine-learning analysis of behaviors such as mimicry, mating, gait, and aggression (22–26). Here we report a successful first step in the machine learning scoring and analysis of fly falls. The results indicate that the YOLOv4 model was able to accurately predict the number of falls per hour for a fly, as confirmed by manually annotated ground truth data. The model identified the falls within 0 to +1 seconds relative to the human annotation based on visual inspection. The delay is perhaps due to the difference between human perception of fall start and end times and the more precise analysis of bounding box coordinates and fall threshold configurations. The model had a false negative rate of 0.8%, and a false positive rate of 1.2%.

Here, a total of 5 young flies were assayed as they died from dehydration/starvation stress, and the frequency of falls per hour was observed to spike 2–4 hours prior to death in each case. A total of 3 aged flies were assayed on normal media until death. These flies exhibited an extended period of increased falls prior to death. A previous study reported that flies subjected to dehydration/starvation stress for approximately 12 hours displayed increased locomotor activity (27), suggesting a possible foraging response. Consistent with that idea, we often observed increased total movement activity in the first few hours of dehydration/starvation stress ( Supplementary Figure 1) (16). By contrast, the spike in falls occurs only after total movement activity has greatly decreased, erratic movement has increased, and the fly is near death, consistent with the conclusion that the spike in falls is due to loss of normal locomotor function, as opposed to some feature of normal foraging behavior.

Future goals include enabling real-time fall analysis by adding a layer of passing frame-wise information to the model in real-time. In addition, increasing the video frame rate may allow for more detailed analysis of fall speed and trajectory and the causes for the fall. The software can generate a list of each fall, including their timestamps and the distance moved in the Y direction. In the future, it may be of interest to ask if the height of the falls might vary with age and/or correlate with mortality. Inspection of the YOLOv4 tracking trajectories suggests that falls may occur when the fly is walking, as well as when it is standing still, and this may be an interesting area for future research. Several drugs, including mifepristone and rapamycin have been shown to increase fly lifespan (28,29), and it will be of interest to determine if these drugs and/or other drugs might reduce aging-related falls. In the future, it may be possible to use these video analysis and machine learning techniques to reveal different types and/or causes of falls.

## Supplementary Material

glaf029_suppl_Supplementary_Tables_S1-S8_Figures_S1-S21

glaf029_suppl_Supplementary_Materials
