# Supplementary material for: Machine Learning Scoring Reveals Increased Frequency of Falls Proximal to Death in Drosophila melanogaster
Source: J Gerontol A Biol Sci Med Sci. 2025 Feb 15;80(6):glaf029. doi: 10.1093/gerona/glaf029 (PMC12066005; doi:10.1093/gerona/glaf029)
Supplement: glaf029_suppl_Supplementary_Tables_S1-S8_Figures_S1-S21 [file glaf029_suppl_supplementary_tables_s1-s8_figures_s1-s21.pdf]

Machine learning scoring reveals increased frequency of falls proximal to death in *Drosophila melanogaster*

Faerie Mattins, Shriya Nagrath, Yijie Fan, Tomás Kevin Delgado Manea, Shoham Das, Aditi Shankar, John Tower

Supplementary materials

Contents

eTable 1. YOLOv4 configurations
eTable 2. Hour 13 timestamps
eTable 3. Hour 14 timestamps
eTable 4. Hour 15 timestamps
eTable 5. Hour 16 timestamps
eTable 6. Hour 18 timestamps
eTable 7. Hour 19 timestamps
eTable 8. Hour 21 timestamps
eFigure 1. Fly falls in dehydration/starvation stress aligned with activity and FDHC.
eFigure 2. Fly falls in aging aligned with activity and FDHC.

eTable 1. YOLOv4 configurations

Hyperparameter	Value	Hyperparameter	Value
Batch Size	1	IOU Loss	ciou
Subdivisions	64	IOU Threshold	0.213
Learning Rate	0.001	Class Normalizer	1
Momentum	0.949	IOU Normalizer	0.07
Decay	0.0005	NMS Kind	greedynms
Angle	0	NMS Beta	0.6
Saturation	1.5	Masks	0,1,2; 3,4,5; 6,7,8
Exposure	1.5	Anchors	Defined in each YOLO layer
Hue	0.1	Classes	2
Burn-in	1000	Num	9
Max Batches	10000	Jitter	0.3
Policy	steps	Ignore Threshold	0.7
Steps	8000, 9000	Truth Threshold	1
Scales	0.1, 0.1	Scale_x_y	Varies for each YOLO layer (1.2, 1.1, 1.05)
		Max Delta	5

eTable 2. Hour 13 timestamps

Hour 13 Ground truth	Predicted	False positives?
4:54	04:55	53:33 No fall
6:50	06:51	59:48 Yes fall
28:29	28:29	
39:49	39:50	
39:53	39:53	
48:21	48:22	
49:02	49:03	
50:55	50:56	
54:42	54:42	
56:45	56:46	

eTable 3. Hour 14 timestamps

Hour 14 Ground truth	Predicted	False positives?
9:57	9:57	12:51 Yes fall
11:52	11:52	
12:55	12:55	
15:12	15:12	
17:27	17:28	
25:55	25:55	
28:46	28:46	
33:25	33:25	
36:08	36:09	
36:21	36:22	
44:45	44:46	
49:31	49:32	

eTable 4. Hour 15 timestamps

Hour 15 Ground truth	Predicted	False positives?
0:29	0:29	11:54 Yes fall
3:44	3:44	24:36 Yes fall
4:58	4:58	
7:07	7:07	
19:59	20:00	
23:49	23:50	
27:01	27:01	
28:26	28:26	
42:50	42:51	
43:45	43:46	
44:25	44:26	
45:51	45:52	
53:39	53:40	
58:05	58:06	
59:38	59:39	

eTable 5. Hour 16 timestamps

Hour 16 Ground truth	Predicted	False positives?	False negatives
1:44	1:45	49:23 Yes fall	6:33
5:29	5:30	54:40 Yes fall	
7:22	7:22		
15:56	15:57		
16:30	16:31		
18:16	18:16		
23:02	23:03		
25:51	25:52		
27:09	27:10		
27:25	27:25		
28:42	28:43		
31:52	31:53		
35:33	35:34		
36:01	36:02		
38:22	38:23		
40:24	40:25		
41:17	41:17		
41:42	41:42		
43:43	43:44		
44:30	44:31		
45:57	45:57		
46:58	46:58		
48:47	48:48		
50:11	50:12		
54:54	54:54		
55:39	55:39		
57:57	57:57		
58:40	58:40		

eTable 6. Hour 18 timestamps

Hour 18 Ground truth	Predicted	Ground truth	Predicted	False positives?
0:11	0:12	32:45	32:46	21:16 Yes fall
0:34	0:35	33:42	33:42	30:29 Yes fall
1:06	1:07	34:08	34:08	00:55 Yes fall
2:46	2:46	35:01	35:02	58:53 Yes fall
4:33	4:33	35:25	35:25	
6:31	6:31	35:29	35:29	
8:10	8:11	37:44	37:44	
9:26	9:26	38:07	38:07	
10:14	10:15	39:38	39:39	
11:02	11:03	39:45	39:46	
12:47	12:48	39:53	39:54	
14:43	14:43	40:01	40:02	
15:12	15:12	40:33	40:34	
15:53	15:53	41:36	41:37	
17:41	17:41	42:32	42:32	
18:52	18:52	43:26	43:27	
19:54	19:55	44:04	44:05	
20:48	20:49	45:11	45:12	
21:49	21:49	47:08	47:09	
22:02	22:02	48:47	48:48	
23:45	23:45	49:01	49:02	
24:07	24:07	50:29	00:30	
24:35	24:35	51:09	51:09	
25:24	25:24	52:28	52:29	
26:39	26:40	54:04	54:05	
27:04	27:04	55:34	55:34	
27:09	27:10	55:48	55:48	
28:10	28:11	55:59	55:59	
28:19	28:20	56:10	56:11	
28:34	28:35	57:04	57:04	
29:20	29:21	58:26	58:27	

eTable 7. Hour 19 timestamps

Hour 19 Ground truth	Predicted	Ground truth	Predicted	False positives?	False negatives
0:06	0:06	38:05	38:05	0:33 No fall	42:28
1:22	1:21	38:28	38:29	4:49 Yes fall	
1:31	1:31	38:39	38:40	9:53 Yes fall	
3:09	3:10	38:45	38:45	10:19 Yes fall	
3:19	3:20	39:49	39:49	16:08 Yes fall	
3:40	3:40	40:12	40:13	16:36 No fall	
5:50	5:51	40:41	40:42	17:48 Yes fall	
7:06	7:06	40:53	40:53	20:14 Yes fall	
7:27	7:27	41:30	41:31	20:37 Yes fall	
7:57	7:57	42:06	42:07	20:46 Yes fall	
10:39	10:40	42:22	42:23	31:18 Yes fall	
11:04	11:05	43:14	43:15	44:47 Yes fall	
12:14	12:15	44:01	44:02	58:36 Yes fall	
14:06	14:07	44:32	44:33		
14:34	14:34	44:42	44:43		
14:44	14:45	46:05	46:06		
15:03	15:03	46:16	46:16		
15:42	15:42	46:38	46:39		
16:30	16:30	46:48	46:48		
19:21	19:21	47:18	47:19		
19:28	19:29	48:11	48:12		
19:50	19:51	48:59	49:00		
21:36	21:37	49:27	49:28		
22:13	22:14	49:46	49:47		
23:21	23:22	50:15	50:16		
23:33	23:34	51:06	51:07		
23:51	23:51	51:23	51:24		
24:00	24:00	51:31	51:32		
24:37	24:38	52:00	52:00		
25:17	25:18	52:14	52:15		
25:52	25:52	52:58	52:59		
26:10	26:11	53:11	53:12		
27:22	27:22	53:35	53:36		
27:45	27:46	54:49	54:50		
28:16	28:16	55:20	55:22		
29:37	29:38	55:28	55:29		
29:55	29:55	55:46	55:46		
30:45	30:46	56:45	56:45		
32:39	32:39	57:34	57:35		
32:47	32:47	57:43	57:44		
34:44	34:45	58:05	58:05		
34:52	34:52	58:18	58:19		
35:20	35:21	58:43	58:44		
36:07	36:07	59:02	59:03		

Hour 19 Ground truth	Predicted	Ground truth	Predicted	False positives?	False negatives
36:16	36:17	59:26	59:26		
37:09	37:10	59:37	59:38		
37:20	37:21	59:42	59:42		
37:25	37:26				
37:54	37:55				

eTable 8. Hour 21 timestamps

Hour 21 Ground truth	Predicted
2:11	2:11
6:13	6:14
7:14	7:14
8:39	8:39
12:51	12:52
49:59	49:59

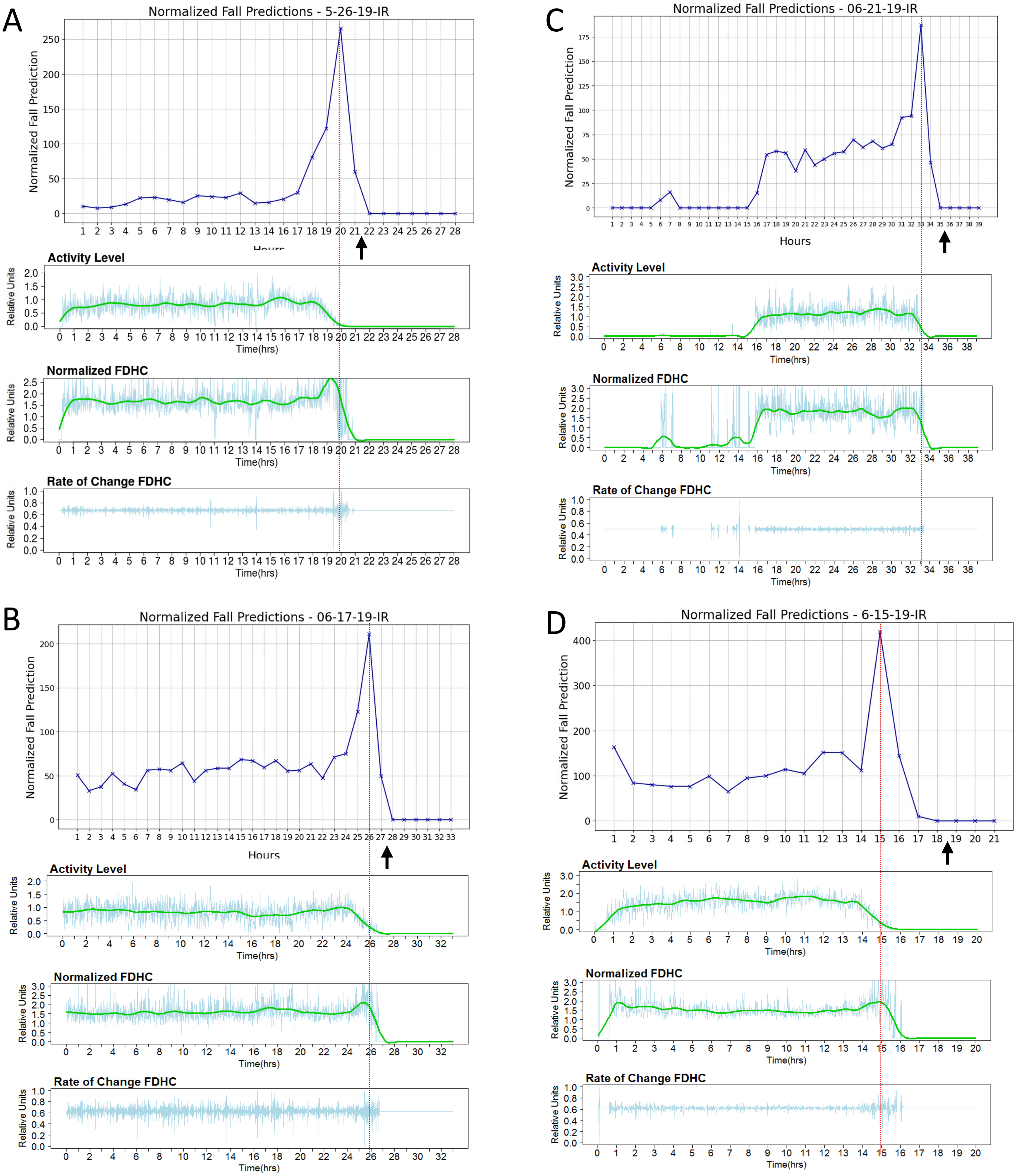

eFigure 1 continued next page.

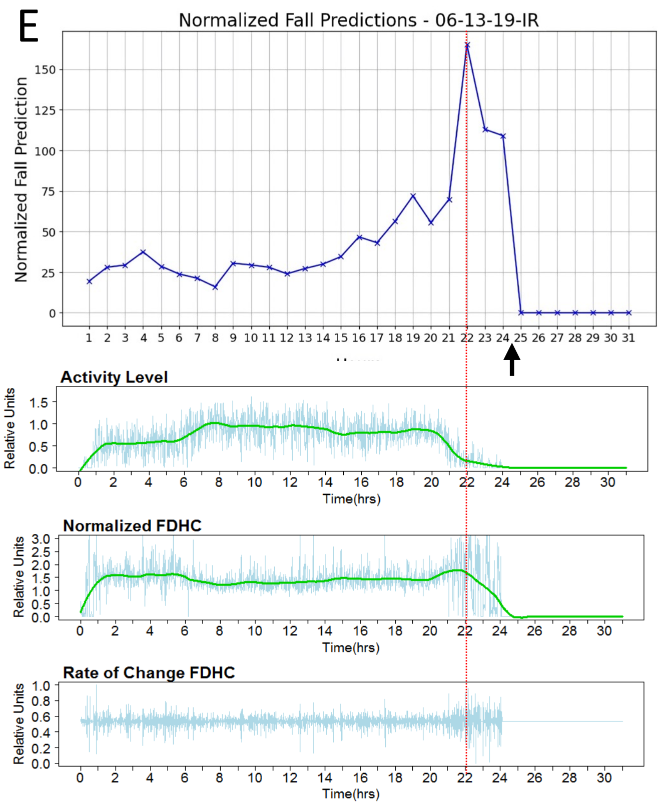

eFigure 1. Fly falls in dehydration/starvation stress aligned with activity and FDHC. Normalized fly falls from Figure 2 are aligned with total movement activity, normalized FDHC, and rate of change in FDHC. Arrows indicate hour of fly death. (**A**) Young (3-5 day old) *w[1118]* male fly (FLYID#5-29-19). (**B**) Young (1 day old) *w[1118]* female fly (FLYID#06-17-19). (**C**) Young (1 day old) *w[1118]* female fly (FLYID#06-21-19). (**D**) young (3-7 day old) *w[1118]* female fly (FLYID#6-15-19). (**E**) Young (14 day old) *w[1118]* female fly (FLYID#06-13-19).

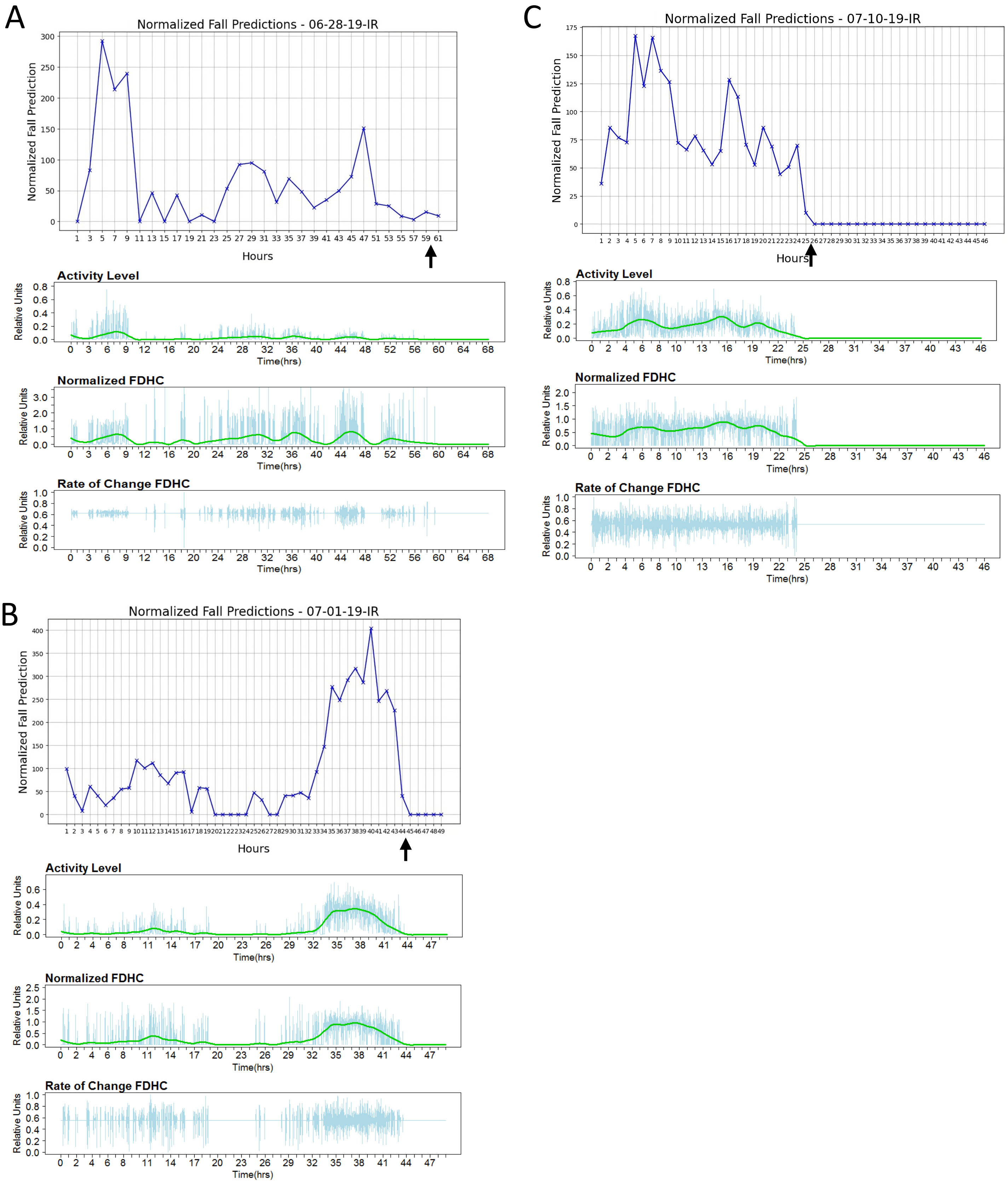

eFigure 2. Fly falls in aging aligned with activity and FDHC. Normalized fly falls from Figure 2 are aligned with total movement activity, normalized FDHC, and rate of change in FDHC. Arrows indicate hour of fly death. (**A**) Aged (64 day old) *w[1118]* male fly (FLYID#06-28-19). (**B**) Aged (66 day old) *w[1118]* male fly (FLYID#07-01-19). (**C**) Aged (75 day old) *3xP3-GFP* male fly (FLYID#07-10-19).
